# Tools in the Investigation of Volatile Semiochemicals on Insects: From Sampling to Statistical Analysis

**DOI:** 10.3390/insects10080241

**Published:** 2019-08-06

**Authors:** Ricardo Barbosa-Cornelio, Fernando Cantor, Ericsson Coy-Barrera, Daniel Rodríguez

**Affiliations:** 1 Biological Control Laboratory, Facultad de Ciencias Básicas y Aplicadas, Universidad Militar Nueva Granada, Cajicá 250247, Colombia; 2 Bioorganic Chemistry Laboratory, Facultad de Ciencias Básicas y Aplicadas, Universidad Militar Nueva Granada, Cajicá 250247, Colombia

**Keywords:** biologically-actives, volatile organic compounds, semiochemicals, chemical ecology, insect interactions

## Abstract

The recognition of volatile organic compounds (VOCs) involved in insect interactions with plants or other organisms is essential for constructing a holistic comprehension of their role in ecology, from which the implementation of new strategies for pest and disease vector control as well as the systematic exploitation of pollinators and natural enemies can be developed. In the present paper, some of the general methods employed in this field are examined, focusing on their available technologies. An important part of the investigations conducted in this context begin with VOC collection directly from host organisms, using classical extraction methods, by the employment of adsorption materials used in solid-phase micro extraction (SPME) and direct-contact sorptive extraction (DCSE) and, subsequently, analysis through instrumental analysis techniques such as gas chromatography (GC), nuclear magnetic resonance (NMR) and mass spectrometry (MS), which provide crucial information for determining the chemical identity of volatile metabolites. Behavioral experiments, electroantennography (EAG), and biosensors are then carried out to define the semiochemicals with the best potential for performing relevant functions in ecological relationships. Chemical synthesis of biologically-active VOCs is alternatively performed to scale up the amount to be used in different purposes such as laboratory or field evaluations. Finally, the application of statistical analysis provides tools for drawing conclusions about the type of correlations existing between the diverse experimental variables and data matrices, thus generating models that simplify the interpretation of the biological roles of VOCs.

## 1. Introduction

Currently, the investigation of volatile organic compounds (VOCs) in ecology relies upon highly diversified technologies for improving the cognizance of the interactions between the various living beings that utilize these molecules, such as plants, arthropods, fungi, etc. From the distinct known interactions, those associated with insects stand out because they are implicated in several decisive processes in agronomic ecology, such as pollination or even the spread of pests. Because of this, the investigations into VOCs related to insect–insect and insect–plant communications and other interactions have gained importance in pest management, from both chemical and biological points of views [1].

The biologically-generated VOC field is widely developed, and thousands of such compounds have been described. Thus, the investigation into this field requires a holistic approach that is achieved by means of metabolomics, which explores the production and concentrations of secondary metabolites from organisms (i.e., metabolomics) and their metabolomes and correlates these variables to biotic activities or environmental conditions [2]. This is an extensively developed and systematized branch of knowledge, utilizing a broad range of methods that consider metabolite collection as a function of their properties and origin [3], followed by analytical procedures, including molecular biology and instrumental techniques [4], down to compiled data matrix management using mathematical tools [5]. From this integration, it is possible to obtain detailed information about the chemical composition of complex natural extracts to unveil their metabolomes and associate them with particular conditions [6].

Among the VOCs biologically active in insects, metabolomics has addressed several semiochemicals such as pheromones (i.e., the chemical communication language of various insects) [7] and allelochemicals (or allelomones) emitted from other organisms. Pheromones are those substances produced by one animal and excreted to perform a specific effect on another individual from the same species, i.e., mediating intraspecific interactions, while allelochemicals are compounds that mediate interspecific interactions, affecting more of the other species than the producing ones. In this sense, allelochemicals are subclassified depending on the adaptive effect on such species involved within the interaction. Thus, allomones have a neutral effect on the receiver but modify its behavior to benefit the emitter; kairomones are favorable to the receiver but not for the emitter, and synomones benefit both the emitter and the receiver. Both pheromones and allelomones are part of a larger group of compounds called semiochemicals [8].

The plant-derived VOCs are very interesting because their nature and production are related to atmospheric conditions [9] and biotic relationships [10]. Molecules coming both from aerial and underground environments have been profiled with the aim of better recognizing the existing communication between plants and organisms in their surroundings [11,12]. Otherwise, insect pheromones have been assessed intensely in metabolomics with a chemical ecology scope, achieving the description of diverse related biological functions of insects, such as mating, aggregation, alarm, social hierarchy and defense. In other words, pheromones have been analyzed in an evolutionary, biochemical and structural background [7,13]. However, metabolomics is not enough for recognizing the explicit effect of a molecule, both singly or in a mixture, on a particular insect, whence such complementary methods as behavioral assays gain relevance for the identification of corresponding responses and allow the inference of biological functions in a defined ecosystem [14]. Therefore, laboratory experiments might be conducted to sample, detect and identify biologically-active VOCs to unravel and explain their roles, prior to understanding the implications in the natural environment and under field conditions [15].

**Figure 1:**
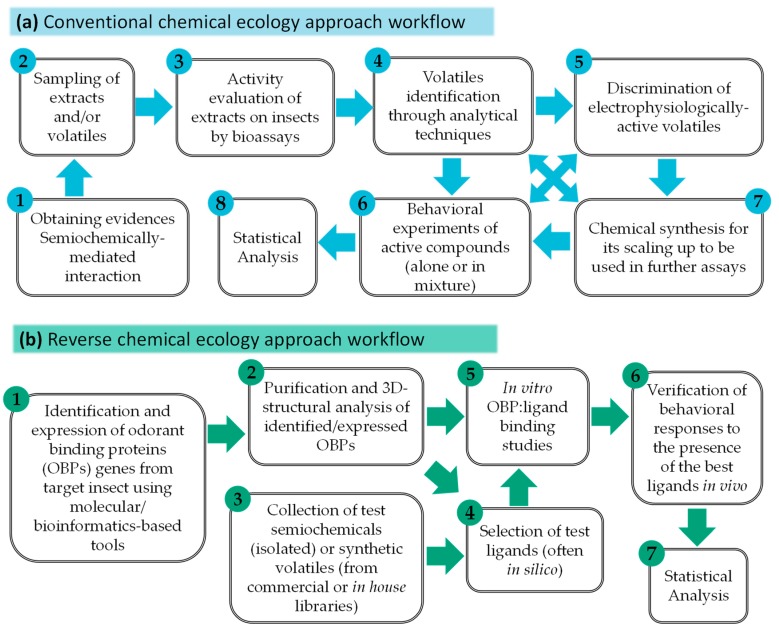
Comparison of workflows of conventional and reverse chemical ecology approaches.

The unification of efforts and results obtained through these techniques has allowed the evolution of chemical ecology related to insect-active compounds so far. Hence, just to cite a few examples, several interesting cases involving complex semiochemically-mediated interactions have been described in previous reviews, such as those microbes-targeting semiochemicals produced by ants to manipulate the soil environment [16], the chemically-mediated defensive behaviors of ground beetles [17], and those chemical cues that regulate/affect insect family and parental care [18]. Additionally, the repercussions of climate change have also been considered in the modification of the corresponding patterns of these remarkable interactions in nature [9,19]. Thus, it is relevant to explore general methods, both practical and analytical, used in the investigation of volatile semiochemicals, which is the scope of this review. Owing to the important advances on understanding the molecular basis of insect olfaction, the reverse chemical ecology approach has recently been raised for the screening of olfactory proteins [e.g., odorant receptors (ORs) or odorant-binding proteins (OBPs)] as a recent, modern approach toward active volatile identification [20]. Figure 1 provides the respective flow diagrams involving the most important steps of both traditional and reverse approaches for studying and identifying active volatile semiochemicals.

In this context, the traditional/conventional workflow for the study of volatile semiochemicals can be generally delimited into the following experimental/conceptual steps: (1) obtaining evidence of the semiochemically-mediated interaction through laboratory/field behavioral experiments; (2) sampling of the volatiles involved in the interaction to afford extracts; (3) evaluation of the activity on insects of the obtained VOC-containing extracts through bioassays; (4) identification of components in extracts by means of analytical techniques; (5) discrimination of electrophysiologically-active volatiles; (6) assessment of the behavioral effect of active compounds (alone or in mixture) in laboratory and field bioassays under correct experimental design and statistics; and (7) if the availability of volatiles is not enough, chemical synthesis can be alternatively performed to scale up the amount to be used in further evaluations. To comply with these steps of the conventional approach, diverse methods, techniques and procedures are then required. Therefore, different tools for sampling and analyzing (detecting/identifying) volatile semiochemicals, as well as bioassays and statistics, are then compiled through published information so far, as an effort to provide a reference material as a practical guide for further studies on insect-associated semiochemicals using the conventional approach. The above-mentioned reverse chemical ecology approach will be briefly described in the section on sensor-based detection of semiochemicals. In addition, some published works using the discussed/described tools are also incorporated, constituting a small sample to denote the plausible scope of the tools under a non-systematic survey, owing to the number of studies using such tools, which are huge and almost countless.

## 2. Tools for Sampling Semiochemicals to Study Insect Interactions

The study of volatile semiochemicals on insect interactions has required the development of very sensitive analytical techniques for their sampling as well as detection/identification, because they are found in very small amounts in natural systems. Sampling of volatile semiochemicals can be based on their extraction (often using solvents) or their collection (air or headspace trapping) [3]. Although it is still recommended to verify that there is a clear response to a chemical signal by means of a biological test, the first step in such studies is to sample the semiochemicals [15]. Thus, after having a good knowledge of the system under study (e.g., evidence of semiochemical production, behavior, morphology, biology, participants, sources, etc.) and a careful selection of different available sampling tools depending on chemical and physical characteristics (e.g., composition, volatility, stability, polarity, etc.), the sampling can be conducted ensuring an adequate semiochemical-containing sample to be used in further bioassays and chemical analyses. The particular collection or extraction tool to be used is dependent on the type of semiochemical(s), the number of insects, and the insect species under study. Below are described the most common tools for extracting and collecting volatile compounds that can be employed within studies of chemically-mediated insect interactions.

### 2.1. Most Commonly Used Methods for Extracting Semiochemicals

For insects, extracting methods are very useful and constitute a practical and rapid way to obtain volatile semiochemicals from those insects having storage organs/reservoirs and/or production glands. If the insect morphology is adequately known, this extraction can be more selective, reducing the risk of contaminants (e.g., fatty acids). Similar information must be previously recorded for other organisms, such as plants (leaves, flowers) or microorganisms (hyphae, colonies), to ensure a more selective extraction. In addition, the extraction yield can be relatively high on combining the resulting extracts after repeated extraction cycles. The basic process of extracting methods is to put the biological material in contact with a solvent (organic or water) that will extract the semiochemicals. Thus, for these purposes, two methods have been most used: solvent-assisted extraction (SAE) and distillation.

#### 2.1.1. Solvent-Assisted Extraction (SAE)

This method is one of the most widely used for VOCs and other metabolites obtained from biological sources in general due to its easy reproduction, versatility in implementation and relatively low cost. The technique consists of contact of a solvent with the biological sample for which extraction will be performed (which can be a whole organism or a portion of the same, such as an organ or tissue). Such a solvent penetrates the matrix and elutes from it with a variable quantity of dissolved metabolites, depending on the involved solubility characteristics. Thus, the metabolite-enriched solvent mixture is then separated from the original matrix affording the crude extract.

The most basic version of SAE is the common solid–liquid extraction, which in turn is the most utilized of the SAE methods for VOC extraction [21,22,23,24,25,26,27,28,29,30]. The extraction effectiveness is variable according to the sample itself and the correct selection of the solvent, and for VOCs, the low polarity organic solvents such as hexane, pentane and dichloromethane are preferred. The results can be enhanced by manipulating physical parameters such as mass transfer (stirring), temperature [3], and surface. A classic example of exploitation of these parameters is Soxhlet extraction, which achieves continuous sample extraction by solvent recycling derived from successive in-situ distillations, allowing the solvent to enrich on compounds from the sample with increasing solubility derived from heating.

Instrumental tools are also available for increasing the SAE efficiency, which manipulate the physicochemical properties of the solvent in a systematic manner. One of these approaches is microwave-assisted solvent extraction (MASE) [3], in which radiation is employed for quick heating of the sample and shortening of the process duration. For MASE, the use of a solvent that absorbs microwave radiation is necessary. One of these other techniques is supercritical fluid extraction, especially with carbon dioxide (CO_2_) for VOC collection [25]. The SFE exploits the attributes of CO_2_ as a supercritical fluid to obtain the best solvent qualities compared to the regular organic solvents, such as greater sample penetration, and once the process has ended, the CO_2_ can be reverted to its gas phase, facilitating its removal by evaporation, with no residues in the sample. Finally, in ultrasound-assisted extraction [25,31], a sound frequency is applied to the material that is being extracted to promote its fragmentation and poration at the cellular scale, then the compounds diffuse more rapidly to the solvent. This is performed at low temperatures to minimize thermolabile compound loss, but isomerization or decomposition of chemically unstable compounds can take place [3].

Some complementary treatments for SAE can be applied with the purpose of pre-concentrating the existing VOCs from the extract prior to the analysis when the concentration of the metabolite of interest is lower than required. For example, a stirring-bar sorptive extraction (SBSE) can be performed on solvents with low VOC concentrations and then the volatile compounds can be recovered using a more affined solvent with successive washing/storing cycles. This method has been shown to be effective in the concentration of VOCs recovered from water and involved in the relationship between plants of *Potamogeton perfoliatus* and *Myriophyllum spicatum* and the aquatic beetle *Macroplea appendiculata*; the concentration of the VOCs in the original extract would be problematic for analysis and difficult to recover using other techniques [26]. Additionally, solvent-assisted flavor evaporation (SAFE) is a vacuum distillation that allows VOCs to separate from nonvolatile substances in the original extract, with successful utilization in the preconcentration of VOCs from insects [30].

#### 2.1.2. Distillation

Some procedures for VOC extraction are carried out under distillation. The classical methods include hydrodistillation (HD), steam distillation (SD) and simultaneous solvent distillation extraction (SDE). In HD, the sample is immersed in water and then heated to ebullition, generating a steam able to break the cellular structures from the sample and pull out the VOCs. At the end of the process, the remainder of the water is extracted with a solvent. In SD, the steam also passes through the sample and pulls out the VOCs, but it is produced in an independent recipient and transferred to the sample. And after this, the steam with the VOCs is cooled using a condenser, which is then collected and allowed to settle for subsequent liquid–liquid separation [25]. In the SDE, the sample is placed on a water-containing recipient with heating, while in another one, the solvent is also heated. The VOC capture takes place when vapor phases of both water and solvent combine, then the solvent with the VOCs is condensed and recovered. Distillations have the disadvantage of being time-consuming and requiring many samples due to their low yield, in addition to being destructive sampling methods and promoting the hydrolysis of some molecules. Nevertheless, they are frequently used in VOC extraction [3].

Due to the disadvantages in cited classical distillation methods inherent to external heating, recently, new procedures have been developed from the concept of internal sample heating. Among these new distillation techniques, microwave-assisted hydrodistillation (MWHD) can be counted [32,33]. With this technique, water, with a sample, is heated directly by using radiation, with incremental extraction yields compared to conventional HD. However, the isomerization of some compounds can be promoted when high power microwaves are used [3]. Another alternative consists of distillation with an ohmic current that electrifies the sample for extraction in water with a buffer by using electrodes and applying potential consistently during the entire process, promoting heating from the inside [31].

### 2.2. Most Commonly Used Methods for Collecting (Trapping) Semiochemicals

The collection of volatile semiochemicals comprises different ways to trap volatile compounds directly in air or headspace from living organisms during defined periods. Thus, all released semiochemicals are deposited onto sorbents (e.g., porous polymers, active fibers or coated materials) and, once collected, they are subsequently desorbed using organic solvents or thermal protocols. The lower level of contaminants is an advantage of this kind of sampling. Therefore, the most common tools for the capture of semiochemicals are described in the following subsections.

#### 2.2.1. Enclosure Techniques

These techniques consist of the confinement of a portion of VOC-containing atmosphere inside a recipient, e.g., canister, cuvette, flask or a special bag. The VOCs collection can, therefore, be performed in passive mode (i.e., no gas flow is employed) or by means of a pump or ventilator, trapping them in a sorbent material, or the container itself. The collected VOCs are subsequently analyzed through analytical platforms. The concept of this method is to keep the VOCs in their original concentrations within the source atmosphere as much as possible until analysis. Diverse factors must be considered for avoiding the loss of some compounds, such as temperature, relative humidity, light exposure, containing recipient surface and sorbent materials, or the control of reactive species (e.g., oxidizers) and therefore ensure reliable measurements [34].

##### Static (SHS) and Dynamic Headspace (DHS) Sampling

The vapor above a condensed phase or surrounding a sample/organism, so-called headspace (HS), contains the volatiles released by the target itself, so it can be sampled and analyzed to provide insights into the composition of this HS. There are two main classifications of HS sampling [35]: static (SHS), in which the analytes on the vapor phase are accumulated passively on capture material by sorption; and dynamic (DHS), which employs a gas flow to assist with the extraction and concentration of volatile compounds on the adsorbent phase [36]. There are a huge number of published cases using DHS sampling, so the collection of VOCs has been achieved, for instance, from whole intact plants [37] or those with controlled herbivory [38,39], flowers [40,41], fresh fruits [42], insect dregs [37], roots [43], and living insects [44]. 

For DHS, there are many materials explicitly designed for this purpose, and their use is common. An interesting application has been reported using the same principle with an alternative device consisting of a portion of a gas chromatography column for the collection of pheromones from *Grapholita molesta*, with results comparable to those from a commercial product SuperQ [45]. A variant of dynamic HS is the purge-and-trap method, which consists of the confinement of a VOC source on a recipient attached via conduits to a pump that passes inert gas through the sample (purging), facilitating the release of VOCs that are then carried to an adsorbent trap where the compounds concentrate. The abovementioned trap is then extracted with the solvent for VOC recovery [46].

##### Sorbent-Based Trapping [Solid-Phase Extraction (SPE)]

Semiochemicals can be passively collected (even at ppm to ppb levels) over a defined period from the HS using small loads (20–200 mg) of a polymeric sorbent packed into a single tube (solid-phase extraction (SPE) from the HS). This tube is made of durable, chemically-inert materials, e.g., borosilicate glass or stainless steel. To improve the trapping, the HS is either pulled or pushed through the packed sorbent at a constant flow-rate (0.1–2 L/min) using a vacuum pump or compressor, respectively [47]. The trapped volatiles are subsequently eluted using low-polar organic solvents (although thermal desorption can be possible) for further analysis typically using gas chromatography (GC) or GC coupled to mass spectrometry (GC/MS). [48]. A common setup of this kind of sampling is known as volatile collection traps (VCTs), comprising the sorbent material packed into a borosilicate glass tube, which is retained with a borosilicate glass wool plug and a PTFE compression seal. 

The type of sorbent determines the semiochemical selectivity during trapping. Therefore, a careful choice of the appropriate sorbent material should be made. The most common commercial options are Tenax (2,6-diphenyl-*p*-phenylene oxide) GC^®^ (60 to 80 mesh) and Porapak (ethylvinylbenzene-divinylbenzene) Q^®^ (80 to 100 mesh size), their refined versions Tenax TA^®^ and Super-Q^®^, and activated charcoal. Other carbon-based adsorbents are graphitized carbon blacks (Carbotrap^®^ or Carbopak B^®^) and carbon molecular sieves (e.g., Carboxen^®^ or Carbosieve^®^) [49]. HayeSep^®^ porous polymers are considered second-generation materials, with minimal shrinkage and monomer bleed, and are interchangeable with the Porapak^®^ series for the collection of volatiles [50]. Each material has its own distinctive fabrication and physicochemical characteristics [51]. For instance, Tenax and Porapak (or HayeSep) sorbents share low affinity for polar and low-molecular weight organic compounds and a high affinity for lipophilic to medium-polar compounds and medium-molecular weight organic compounds, but the thermal stability is different (350 °C and 250 °C, respectively), Tenax being more appropriate for thermal desorption [52]. Additionally, the amount of sorbent material is another important factor to take into consideration for VOCs collection. Such a load depends on the chemical features of the VOCs to be collected, adsorbing capacity of the sorbent, the sampling volume, and the gas flow rate [47,53].

##### Solid-Phase Micro-Extraction (SPME)

SPME is a non-exhaustive technique that isolates and concentrates volatile analytes on a miniaturized extraction phase (a polymeric coating material as sorbent) from a matrix/medium in a liquid or gas phase in one step without the intervention of any solvent. In the process, the volatile molecules enter into contact with the sorbent material and deposit onto its surface by intermolecular interactions, with strengths that depend on the affinity of the material for the volatile molecules. This method is primarily useful for VOC collection for qualitative purposes; although quantitative analysis can be performed, a calibration curve is required for each compound using appropriate standards for absolute quantification (see more details in Section 3.1.1) [51,54].

The efficiency of VOC capture by SPME depends on assorted variables, including the coating material itself, which is probably the most important factor because the diversity of available compositions and dimensions is broad and these parameters determine the affinity for specific groups of molecules. If possible, to enhance the choice of the best option, a preliminary evaluation of different coatings is advisable, considering their performance in the recovery of desired metabolites through thermal or solvent desorption. In the first case, the metabolites are desorbed directly from the material with a high efficiency and can be coupled directly to GC, but desorption must be performed quickly and below the decomposition temperature of the sorbent to avoid permanent damage. In solvent desorption, the main advantage is the possibility of storing the dissolved VOCs for ulterior analysis or bioassays, but some of the original compounds can be lost due to solubility in addition to the masking of most volatile VOCs by the solvent [51,54].

Apart from the adsorbent selection, there are more variables that intervene in the extracted VOC profile and should be controlled as much as possible, including the humidity range, biological sample uniformity, temperature, and device geometry. Among these, the exposure time is a parameter that is convenient to normalize depending on the experiments, since short periods result in low concentrations of captured compounds, but long periods favor fixation of less volatile compounds while more volatile compounds are desorbed, according to the affinity between adsorbent-VOCs [54,55].

A SPME device is comprised of a protection layer, coating material and support [56]. Currently, different device geometries have been developed to improve the sampling and extraction such as fibers (polymeric coating supported onto a fused silica rod); in-tube (a tube internally coated with extraction phases); and other coated supports such as particles, fabric/blade and stir bar [55]. The first applications of SPME involved fiber geometries [57], and in-tube devices were introduced, which are attachable to analytical platforms for direct desorption and analysis of VOCs. Regarding coating polymers, the most popular are carboxen (CAR), polydimethylsiloxane (PDMS) and divinylbenzene (DVB), both as single or combined materials in different proportions, and polyethylene glycol (PEG), with a broad range of applications due to their distinct polarity [e.g., 7–100 μm PDMS (non-polar), 60 μm PEG (polar) and 65 μm PDMS-DVB (bipolar)] extraction mechanism [absorbents (e.g., PDMS) or adsorbents (e.g., PDMS-DVB)] [55,58,59]. Thus, the correct selection of the polymeric material can enhance the affinity for a particular group of semiochemicals depending on the polarity and/or the analyte size [60]. From commercial options, the main advantages are interlaboratory validation, variable geometry (fibers, film-coated bars, membranes, tapes, etc.), biocompatibility (useful for in-vivo and in-vitro sampling) [61], and applications in diverse fields of knowledge including metabolomics. However, among the disadvantages are the low maximum operating temperature (240 to 280 °C) and low selectivity [55].

##### Headspace—Solid-Phase Micro-Extraction HS-SPME

When the extraction of VOCs of interest is performed through SPME from a gas phase and the adsorbent is located above the emission source without direct contact with it, this is known as HS-SPME [62]. It is very useful for field or in-vivo sampling, which is a substantial advantage considering that it allows the extraction of VOCs that can only be obtained from living organisms in very specific biological or activity-related conditions. This collection can be performed during some particular times of the day or the year and, in this way, it minimizes the loss of these compounds associated with destructive sampling methods. The utilization of adsorbents in fibers designed for this purpose is very common, but recently, other approaches have been considered, such as HS with stirring-bar sorptive extraction (HS-SBSE), in which the adsorbent material is coated on a stirring bar. This technique shows better efficacy when compared with classical HS-SPME because the quantity and surface of the adsorbent are higher [63]. Once the time of capture has elapsed, the bar is washed with distilled water, dried and then submitted to direct thermal desorption in a gas chromatography device [64]. HS-SPME sampling has been a widely utilized method for collecting VOCs from different materials, such as grains at different conditions, as healthy grains [65], grains damaged by insects [66], grains infected with fungus [67,68] as well as from nectar [69], nuts [70], intact leaves or those affected by herbivory [71,72,73,74], bark [75], flowers [41,73,76,77], essential oils [78], roots [79], fresh fruits [80], insect excrement [74], whole plants [81], living insects [82], and more examples which can be found in the available literature.

##### Other Enclosure Technique Devices

The enclosure method does not always involve the trapping of VOCs on adsorbent materials at the time of sampling, and the recipient containing the VOCs can be used for its storage until analysis. Depending on the employed configuration, VOCs can be analyzed by attaching the container directly to the instrument without further sample treatment. Nevertheless, in some cases, an additional procedure prior to analysis can be required, for example, when VOCs are collected for subsequent GC analysis, cryofocusing can be necessary. This consists of cooling the VOCs sample under ambient temperature, aiming to avoid the troubles related to injection peak band distortion [83].

Among the existing options for enclosure without adsorption, sampling bags are widely used due to their economic cost for collecting numerous types of volatile compounds, including VOCs emitted from green plants. However, its disadvantages are the possibility of a leak, short-term storing due to chemical sample instability, and poor recovery for non-polar species. Another alternative is the stainless steel canister (SS canister). The sample is introduced under pressure into a canister coated with inert inner-wall material, allowing sample storage for weeks [84]. In addition, cuvettes manufactured in glass are suitable devices for enclosure without sorption due to its chemical inertness, but configurations including sorbent materials are more frequent. Further information about the cited and other enclosure techniques and examples used to collect biogenic VOCs can be found in a previous work [34].

#### 2.2.2. Direct-Contact Sorptive Extraction (DCSE)

When adsorbent material is in direct contact with the source of the VOCs, the technique is called DCSE and allows for the collection of volatiles from the sample interface and its surroundings. This is a seldom-explored method in studies related to VOCs released in plant interactions with other organisms, but it has recently gained attention in the area. First, replication is not limited by equipment, collection time or weather conditions, which differs from the HS sampling methods. It has been demonstrated that the robustness of DCSE is higher than that of DHS, while at the same time being more sensitive, capturing a broader range of VOCs and being used for the recognition of the effects of herbivory in the field, especially in difficult-to-access areas [63]. It also allows for the possibility of performing in-vivo sampling, and it is reproducible, simple, executable on different plant parts at the same time. This method also preserves intact the plant material to be used in further assays [85].

Within the diversity of described methods for performing a DCSE are the use of stirring bars coated with PDMS as reported by Kfoury et al. (2017) [63]. These stirring bars were located on specific areas of tea leaves infested with *Ectropis obliqua* larvae with the purpose of comparing the difference between the VOC profiles emitted in healthy leaves and those sprayed with methyl jasmonate, finding an improved detection of compounds associated with herbivory and significant reductions in the amounts of emissions from plants under different treatments. Another approach is direct contact sorptive tape extraction (DC-STE), which is implemented by the placement of non-adhesive PDMS tape segments over a biologically active surface and has proven to be helpful in the topographic characterization of VOCs from a plant exposed to different types of stress [85].

## 3. Instrumental Analytical Techniques

Once volatile sampling is carried out, the next step is to analyze the VOCs by means of appropriate methods according to desired objectives. In investigations guided to the assessment of VOCs with biological activity or response in insects, two fundamental aspects are integrated: the first is the profiling of collected compounds, i.e., VOCs, and their general characterization, and the second is the full identification of the biologically active components. The majority of the required procedures for performing an accomplished work on these conditions draw on instrumental analytical techniques, which offer many advantages in VOC characterization. Here, some of these methods are described.

### 3.1. Gas Chromatography (GC)

GC is an instrumental technique of separation and analysis focused on volatile compound-containing samples. The partitioning of components is possible when the sampled VOC mixture is introduced at the beginning of a chromatographic column, and the mixture is passed through, aided by an inert gas flow. Then, the components interact differentially with the inner column material at the molecular scale according to the compound-column affinity, which is specific for each one at fixed conditions, promoting their separation. Thus, the components are singly eluted from the column for immediate detection and analysis of compound-specific signals. The process is optimized by temperature control, either with constant values or programmed increases.

GC is the most commonly used technique for VOC analysis because it allows simultaneous separation and analysis of volatile components with great sensitivity, robustness, reproducibility, and versatility. This technique is suitable for thermally stable volatile compounds since high temperatures are used for sample vaporization and in the chromatographic separation itself [86]. For analysis, GC requires detectors for the recognition, identification and quantification of separated components and allows for a wide variety of them. The most convenient detector choice depends on the compound’s physical and chemical properties, origin and concentration as well as the scope of the analysis, etc. In the investigation of VOCs with biological activity on insects, the detectors used with more frequency are the mass spectrometry detector (MSD) for molecular identification of compounds [6,86], flame ionization detector (FID) for quantification, and the electroantennography detector (EAD) for in-line analysis of biologically active molecules.

In addition to the separation role of GC, the chemical identity of volatile semiochemicals can be achieved from retention data. Accordingly, retention indices are well-recognized as a significant parameter to the VOC identification within a standardized system [87]. The common process for this identification comprises the calculation of retention indices from retention times (t_R_) of each resolved compound in the GC profile. This calculation is based on the equations proposed by Kováts [for isothermal GC analysis, called retention index (I) or Kováts index (KI)] and van den Dool and Kratz [for programmed GC analysis, called the retention index (I), linear retention index (LRI) or programmed-temperature retention index (PTRI or IT)] [88]. KI and LRI do not have the same meaning, and usually in the literature they are misused or exchanged, so such a difference should be considered depending on the GC analysis. In the equations, the retention trend of VOCs is related to a uniform scale determined by a homologous series, having a linear relationship between the Log(t_R_) and the carbon number, such as n-alkanes, 2-alkanones, and linear fatty acid methyl esters, among many others [89]. These calculated RI values are related to the published information or databases; however, to ensure reliable identification, the RI calculations must be performed on retention data obtained from different runs using at least two GC capillary columns, involving a non-polar stationary phase [e.g., HP-5^®^ (5%-phenyl)-methylpolysiloxane)] and a polar stationary phase [e.g., carbowax^®^ (polyethylene glycols)]. A detailed compilation of the scope and characteristics of the use of LRI was published by Zellner et al., (2008) [88].

A larger number of peaks can be detected if the chromatogram is recorded by more than one GC column. In this sense, a vast advancement has been reached with the technique so-called comprehensive two-dimensional (2D) gas chromatography (GCxGC) [90]. The mixture is firstly separated on a high-resolution capillary GC column and resulting fractions are refocused and injected into a second GC column. A modulator (an interfacing device) connects both columns and discriminates the eluate from the first column into the subsequent small fractions, having no larger than one quarter of the peak width to ensure separation [91]. Both columns can be held in the same oven (identical temperature programming conditions), although other setups involve two different ovens for each column (different and flexible temperature programming). A low- or intermediate-polar phase containing GC column (typically a 15–30 m × 0.25–0.32 mm I.D., df 0.1–1 μm) is usually used in this kind of analysis, employing temperature programming (heating rate = 1–5 °C/min) and longer times (45–120 min), affording the first dimension. A shorter, narrower GC column (having a highly polar phase and a shape-selective nature), is then used as the second one (typically 1–2 m × 0.1 mm I.D., df 0.1 μm) during an extremely fast analysis (1–10 s) [90,91]. The resulting chromatogram is a 2D-plot involving the retention times (in min) after first-column (first dimension) and second-column (second dimension) separations. Hence, the overlapping peak reduction and minor/trace compound detection can also be achieved with this technique. These facts are very important improvements, since minor/trace compounds would be undetected in one-dimensional GC, particularly if bigger peaks overlap such constituents. Results using GCxGC are extremely promising and it will become the technique of choice for GC analysis of complex mixtures during semiochemical identification owing to the separation efficacy of this technique [92].

#### 3.1.1. Gas Chromatography Coupled to Flame Ionization Detectors (GC-FID)

The FID is the most recognized detector for GC because it is very sensitive to volatile compounds with hydrocarbon-based structures and has a mass flux-dependent response, which in turn makes it very feasible for VOC analysis [93]. This detector is very popular for quantitative purposes, after the building of calibration curves (i.e., peak areas versus concentration plots) for each compound, but is not sufficient for accurate compound identification by itself, which is overcome by the use of respective standards (co-chromatography), using linear retention indices in comparison to the information in databases, or working with well-characterized profile samples. Therefore, the analyses of compounds biologically active on insects that employ GC-FID include an additional detector to complement the VOC profiling of a sample, such as the mass spectrometry detector (MSD) [94]. 

The determination of absolute amounts of semiochemicals using GC-FID after sampling is possible if the analytical procedure is entirely standardized (sampling and chromatographic analysis) with appropriate quantitative standards for reliable comparisons and validated results [95]. Additionally, some analytical precautions must be taken into consideration if SPME is used for collection to ensure a correct quantitative analysis, since the SPME recovery rate of any individual compound is different as well as volatiles can compete against each other [96]. In this regard, a mixture of the respective standards should be prepared in such relative proportions similar to those presented in the target samples, to be included into internal and external calibration procedures. Only in this way, the GC peak areas can be used to calculate absolute quantities of semiochemicals from SPME samplings [97]. On the other hand, a comparison of relative quantities (using GC relative abundance data) of a particular volatile across samples/treatments/species after SPME (if collected and analyzed under identical conditions) can be performed, but such a comparison of different volatiles within a chromatographic profile is not possible owing to the above-mentioned reasons related to the SPME itself.

Some examples of recent investigations within the bulk of studies using GC-FID are the identification of sex pheromones of pest insects [46]; specific parasitoid-attracting molecules [37]; volatiles released by plants in the presence of a pollinator [98]; the assessment of alteration in pest response to plants infected with phytopathogens [67]; assessment of essential oils [99], some of which performed fumigant and repellent activity [100], as well as in those studies involving plant VOC profiling [75,101]. Although plant VOCs are not usually profiled in the context of plant-insect interaction, provides valuable data for further investigation regarding this topic.

#### 3.1.2. Gas Chromatography Coupled to an Electroantennography Detector (GC-EAD)

In this technique, the compounds partitioned in chromatographic columns are analyzed on an EAD, which consists of two electrodes (one for recording, and the other one for reference) connected to a recently excised insect olfactory organ (antenna), head, or even the whole body from alive or sacrificed individuals [13]. When active VOCs touch the biological receptor, an electric potential difference is produced as the compound passes through with a signal commensurate to the concentration and response factor that is produced in the insect, and for compounds with no stimulating effect, the electric potential remains without remarkable alterations. The electric potential difference produces a current that is measured by an electrode, and then an electroantennogram is generated.

When a GC-EAD is utilized for VOC profiling as a function of antennal response, the real-time recognition of chromatographic peaks having such an effect can be achieved [102]. Nevertheless, the chemical identity of profiled compounds cannot be determined when using this method separately, and for this reason, it is common that the EAD is used in complement with FID in split configuration. Additionally, EAD does not provide accurate information about the type of effect that a compound produces on a live insect because it only shows whether an olfactive response is generated. The utilization of GC-EAD has been intensively used since the 1980s, and some examples include the investigation of pollinator-attractive compounds [21,41], parasitoids [37], pests [67], and the identification of pheromones in complex extracts [29].

#### 3.1.3. Gas Chromatography Coupled to Mass Spectrometry (GC-MS)

GC-MS is a hyphenated instrumental technique that combines the separation attributes of GC with the sensitivity of MSD, making it suitable for analyzing low molecular mass and mid- to low-polarity compounds [103]. It is widely used for VOC identification because it has the advantage of rapid compound identification by comparing the analyte mass spectrum to those recorded on databases using specialized software that indicates the matching results, with high efficiency in recognition of molecule identity in the majority of the cases [104]. Nevertheless, this approach is not very frequently used for VOC quantification in complex samples because a calibration curve for each compound is necessary owing to the variation of the response factor from each analyte in the MSD [86].

The sensitivity of GC-MS is mainly determined by a particular MSD device denominated as an analyzer, which separates and classifies the ions generated from components in groups according to their mass/charge ratio (m/z) [105]. From the distinct commercially available GC-MS analyzers, the most commonly used is the quadrupole, given its low cost and sufficient precision for compound identification. The employment of GC-MS has been reported in several studies such as the characterization of essential oils (some of them having activity on insects) [99,106], metabolic profiling of plant VOCs emitted under different conditions [107,108,109], characterization of natural products with insect-repellency [110]. In addition, GC-MS has been also used for the investigation of plant VOCs related to some insects of interest such as pollinators [98,111], phytophages [101,112], parasitoids [37,113], hosts [26] or vectors [67]. The study of VOCs emitted by insects has also required GC-MS analysis [27,28,30,46,114].

When comparing the proportion of recent publications on VOCs active on insects that reference the use of GC coupled to single-quadrupole mass spectrometer (qMS) against other analyzers, for the latter, the number of citations is lower. Among these, the utilization of ion trap mass spectrometers (itMS) is referenced in studies addressing VOCs related to insect attraction in flowers [69] and whole plants [73], phytopathogenic vectors [115], and parasites [42]. In parallel, the magnetic sector analyzer has been reported for real-time monitoring of insect pheromone release [44] and time-of-flight (TOF) analyzers in the assessment of biosynthesis of VOCs important for pollinator attraction [21], in the characterization of volatile secretions from *Graphosoma lineatum* [116], or in the profiling of compounds present in *Tilia* spp. nectar, which allowed to discard the assumption of the toxic character of nectar for bumblebees [117], among other studies.

### 3.2. Mass-Spectrometry not Coupled with GC

MS is not exclusive to GC because it was an independently created technique; thus, in its evolution, various inventions disconnected from GC have been made, and it is remarkable that despite this fact, MS has contributed immensely to the investigation of key VOCs in this field of knowledge [118]. MS consists of an instrumental method that measures the mass of molecules or fragments from these by means of the mass-to-charge ratio (m/z) when ionized using an apparatus denoted as a mass spectrometer. This mechanism consists of three main sections: the ionizer (where the molecules are converted to particles with electrostatic charge), the analyzer (device that filters, separates and groups the charged particles according to their m/z), and the detector (in which the m/z from the particles coming from the analyzer is quantified) [119].

Some of the most interesting applications of MS in research related to VOCs originating from insects are the superficial scanning of exoskeletons by DAPPI-MS (desorption atmospheric pressure photoionization mass spectrometry) aiming to characterize VOCs for defense produced in the glands, namely, from *Prorhinotermes simplex* soldier termites and from the beetle *Graphosoma lineatum*, achieving a topological picture of emissions [120]. This scope was also employed on *Drosophila melanogaster* with AP-SMALDI (atmospheric pressure scanning microprobe matrix assisted laser desorption/ionization) for metabolite characterization [121] in which the study was addressed to hydrocarbon-chain compounds and there was interest in pheromones with such structure. Other useful applications of MS have been reported for aphrodisiac pheromones of the small cabbage white butterfly (*Pieris rapae*) for release monitoring by means of cDART-MS (confined direct analysis in real-time mass spectrometry) [44], which is a portable MS method and is ideal for assessment in field work [122].

In the investigation of VOCs originating in plants, a remarkable example is a study that employed direct ambient corona discharge ionization mass spectrometry for the characterization of volatiles coming from flowers of selected species (*Photinia serrulata*, *Castanopsis sclerophylla* and *Stemona japonica*) with insect semen-like odor without any further sample preparation. Such research found some compounds that were not previously identified by GC-MS for the species under investigation [123].

### 3.3. Nuclear Magnetic Resonance (NMR)

The current state of VOC investigation is so widely documented that the use of tools associated with compound databases is usually satisfactory for profiling compounds, in addition to complementary chemotaxonomic knowledge. However, previously uncharacterized compounds can be found, and it is important for an entire structural elucidation of the compound to be carried out, which is possible by means of NMR, which provides highly precise information on the structural properties of small molecules.

The basis of NMR consists of the application of a high-power magnetic field to a sample that is often highly purified; then, this field interacts with atomic nuclei that have a spin distinct from zero (for example, ^1^H, ^13^C, ^15^N, etc.), promoting a change in their orientation. When the nuclear spins have a parallel or antiparallel orientation with respect to the external magnetic field, a magnetic pulse is applied for a time and then removed, so the nuclear spins return to their initial orientation, which produces a radiofrequency emission that is conditioned not only by the magnetic field but also by the nuclei chemical environment, i.e., the neighboring atoms’ spatial distribution and type. The NMR equipment then measures the sample emitted radiofrequencies and compares them to corresponding values of the induced magnetic field, registering a spectrum that shows a signal-pattern with values denoted as “chemical shifts” that tend to appear in well-defined ranges according to their corresponding functional group and experimental conditions. The information is further interpreted to correlate the nuclei to each other, and in this way, the whole chemical structure may be deduced [124].

In the investigation of naturally occurring volatile compounds, NMR is usually separated from other instrumental techniques, such as GC, even when GC systems coupled to NMR (GC-NMR) are currently available [125]. This is because of the NMR sample requirements, which often require high purity, as well as compared to other detection techniques (e.g., GC-MS) where a higher amount of sample is needed, i.e., in the order of micrograms. In natural sources, VOCs are often found in lower quantities and there is often interference from other compounds present on the matrix, which makes their purification, recovery and analysis [126] challenging. As stated by Reitz et al. (2015), “NMR is a valuable technique for structure elucidation but suffers from low sensitivity” [127].

Therefore, the application of NMR in topics related to VOCs that are biologically active on insects has been widely referenced for structural elucidation after the isolation of VOCs. Such cases can be exemplified (but not extensively) by the evaluation of desert locust (*Schistocerca gregaria*) secretions [127] and the compounds present in the venom of the little black ant (*Monomorium minimum*) [128], and can be used as a tool for the identification of pheromones from different insects, such as the tea moth [129], *Sitophilus oryzae* [130], *Ectropis obliqua* [131], and the azuki bean beetle (*Callosobruchus chinensis* L.) [132]. In the field of plants, the investigations are much more extended regarding structural elucidation and the identification of purified compounds from essential oils with activity on insects [133,134,135] and the analysis of isolated volatile compounds from *Citrus australasica* [136] and *Punica granatum* L. [137].

## 4. Chemical Synthesis

Today, instrumental chemical analysis is very developed and allows the investigation and recognition of VOC molecular identities using a low-mass amount (nanogram scale) of the compound, which represents a key advantage because natural volatile compounds are often found in very low concentrations. However, these quantities are not sufficient for recovery and utilization in other assessments, thus limiting, in many cases, the experimental size and scope of behavioral experiments, when VOCs are extracted. For this type of situation, chemical synthesis can sometimes be a feasible option, depending on the desired molecular structural complexity and the available reagents.

A remarkable achievement in VOCs obtained by means of chemical synthesis is related to insect pheromones. Several examples in the literature include the stereospecific synthesis of semiochemicals, such as bombykol (the first isolated insect sex pheromone) [138] or (+)-sitophilure, an aggregation pheromone from two species of the *Sitophilus* genus [130]. A few examples within the plethora of studies on semiochemical synthesis are shown in Table 1. In the case of VOCs identified from plants, most of the VOCs involved in ecological interactions can be challenging due to the structural complexity of several molecules, but for some other functional groups, synthesis can be made more easily, for example, a linear hydrocarbon present in the kiwi (*Actinidia chinensis*) floral scent [40] and an ester found in Australian finger lime (*Citrus australasica*) [136].

## 5. Recognition of Biologically Active Compounds

As indicated in the section on GC-EAD, for a complete characterization of activity, it is not enough to carry out the chemical identification of VOCs present in a sample profile, but it is also necessary to perform assays that aid in the analysis of the effects from such compounds on the olfactory structures (including specialized organs or molecular receptors) or live insects, with the aim to observe the response derived from a VOC stimulus, and in this way, establish the corresponding effect and required dose. Apart from the already discussed GC-EAD, there are other available methods for this objective.

### 5.1. Electroantennography (EAG)

The EAG can be performed independently from GC, and in this case, the use of pure compounds is recommended when the objective is the measure of the required dose for generating a response in the insect, admitting that it can also be used for assessing an odor complex or raw sources, both natural and artificial. The functional principle is the same as that described in the GC-EAD section, except that compounds are not conducted from the output of a GC device but are inserted in a controlled manner to the instrument by pumping the odor from a reservoir.

This technique has been used in several studies such as the detection of VOCs emitted by some plants (mainly at early flowering phases) to be active to *Apolygus lucorum* [143], the measure of the olfactory response from adults of *Carpomya vesuviana* to VOCs identified in the fruits of their host (i.e., the jujube) [80], and the comparison of the olfactory responses from adult females of *Trissolcus basalis*, a parasitoid, to floral carboxylic acids from buckwheat, a nectar-providing plant used by the adult female of this parasitoid for feeding [1]. In addition, the dose-response curve for different compounds has been determined and characterized for a variety of insects, such as the moth *Plodia interpunctella* in response to synthetic standards of VOCs emitted by fungus-infected wheat grains [67], as well as in the evaluation of the response of female *Holepyris sylvanidis* ectoparasitoids to volatiles identified in the feces of *Tribolium confusum* larvae, the corresponding host [37].

### 5.2. Single Sensillum Recording (SSR)

EAG must be generally interpreted as a qualitative indicator of olfactory response. However, quantitative measurements of olfactory response can be obtained via single sensillum recording (SSR) [144]. The sensory hairs called “sensilla”, which cover the surface of olfactory organs, carry the olfactory sensory neurons (OSNs). The odorants present in the environment enter the “sensilla”, going inside through pores, and dissolve into the liquid medium denoted as “sensillum lymph”, in which the OSNs are embedded. The odorant receptor proteins (ORP) located on the surface of the OSNs interact with the odorants in the way of odor-gated ion channels, inducing neuronal activity via fluctuations in the basal firing rate of the OSNs. Extracellular recordings from single OSNs can be obtained by placing a recording electrode into the sensillum lymph, while a reference electrode is located in the lymph of the insect body or its eye. The SSR allows for monitoring those differences in potential between the sensillum lymph and the reference electrode, as the receptor activity of the OSN induces the generation of electrical spikes. The number of spikes is variable depending on the odorant, determining a mechanism of odor coding [145]. This tool has been used in several studies such as the demonstration of the role of palps in locusts as olfactive organs [146], the detection of neuronal responses of bed bugs to semiochemicals [147], the evaluation of electrophysiological responses of olfactory receptor neurons in trichoid sensilla of the diamondback moth *Plutella xylostella* [148], the studies on odor coding in *Drosophila* [149] and *Helicoperva* [150], the analysis of olfactory response in *Apis mellifera* [151], and the evaluation of odor responses to morphological classes of sensilla in *Drosophila* [152].

### 5.3. Behavioral Experiments

These are fundamental assays for the identification of compounds biologically active on insects because they allow for the determination of the type of response that a VOC (singly or in mixture) generates on a test organism. This type of experiment permits the utilization of pure compounds or complex sources, such as flowers, green plants, other animals, etc. To ensure the net olfactory response in the experiments, it is typical that the emitting sources are visually isolated from the insects, i.e., the contact with the odor source by insects must be avoided at all times because this could add a factor into the selection and bias the final answer. Additionally, the surroundings and local light sources should be controlled.

Behavioral experiments are adaptable, and a wide variety of designs with variations in volatile release methods have been reported in insect studies, exposing the insects to the VOC source in a controlled manner and minimizing the interference of foreign emissions and other factors such as visual stimuli as much as possible. However, visual stimuli are sometimes required to get olfactory responses (i.e., the well-studied optomotor anemotaxis). Hence, it is suspected that the response of the insect depends on the joint effect of visual and olfactory stimuli, which require specific methodologies [153]. Some configurations consider only one source, while there are other structures that use two or more emission sources, and the corresponding interpretations of results are performed according to the features of the case. 

Within the plethora of studies performing semiochemically-mediated behavioral experiments on insects, those related to moths (mainly males) are the oldest and most studied, since olfaction is very crucial in sex behavior of moths [154]. In this sense, female moths emit sex pheromones which can be detected by conspecific males to find/recognize their mates. Therefore, the pheromone-mediated communication in moths has been an important topic in chemical ecology for several purposes, specifically for understanding those mechanisms of perception/orientation of sex pheromones in males as well as their production/releasing by females [155]. The first sex pheromone was discovered in the female pheromone gland of *Bombyx mori* (silkmoth) as a prevalent 10:1 mixture of bombykol (10,12-(*E*,*Z*)-hexadecadien-1-ol) and bombykal (10,12-(*E*,*Z*)-hexadecadien-1-al) [156]. From such a finding, huge information has been generated to explain/clarify the molecular and neural mechanisms that mediate chemoreception and processing in this insect (and others) during the recent decades [157], but also on understanding the male moths orientation by behavioral experiments. In this regard, several studies are related to the orientation behavior through different movements to enhance the contact probability with the air bearing that pheromone [158], referred to as a plume (i.e., not only smooth dispersals of odor gradients, but sparse disseminations of highly-concentrated packages of air-dispersed odor). Therefore, the odor plume is also an important factor to provide the directional information required for moths (and other insects) to orient toward the odor source [159]. This location, and the subsequent navigation, need both the semiochemical signal and air-borne semiochemical-containing plume [160]. Thus, the bulk of studies has been published involving different experimental setups and devices to investigate the responsiveness to semiochemicals in moths, which is a very important decision in order to have a suitable, reliable measurement of the response [161].

#### 5.3.1. Linear Olfactometry

Linear olfactometry consists of diffusing the test odors through a transparent straight tube in which the organism is located and then monitoring displacement and behavior as a result. Several examples of linear olfactometry-based assays have been described, for instance, the behavioral study of adult individuals of *Cydia pomonella* when exposed to apples under different conditions (healthy, mechanically damaged or infested with larvae of the same species) using linear olfactometry for model exemplification. Such a device was used in order to demonstrate that an insect’s behavioral responses can be predicted after building the relationship between the chemical composition of plant volatiles and the behavioral response of insects using statistical modelling, e.g., generalized linear modeling (GLM) [5]. Additionally, the evaluation of the effect of selected potential semiochemicals (both pure and in mixture) on a group of dog ticks (*Rhipicephalus sanguineus*) was also performed [162]. In comparison to a Y-tube olfactometer, three compounds induced strong responses using a linear olfactometer, where turning and movement (speed, direction and duration) were measured after volatile compounds exposure.

#### 5.3.2. Two-Path Olfactometry

The following method is among the most popular configurations in olfactometry: two independent reservoirs, one containing the VOC source and the other containing the control, are connected to a convergent point by employing an accessory in a T or Y shape using the two closest entrances to each other, and the insect is placed in the remaining point and then allowed to make a choice. One individual is deployed at time, and the path of its election (VOC source or control) is recorded; this operation is repeated several times to achieve statistically significant data, and this is used for assessing both attractive and repulsive effects of VOCs on insects. This approach has been employed in the identification of either attractive or repulsive VOCs from certain plants to the coffee berry borer (*Hypothenemus hampei*) [163] and for evaluation of the influence of VOCs emitted by plants in different states on aphids [72], pollinators [111] or parasitoids [113]. Additionally, this has been used in the study of the effect of plant-derived VOCs on female *Grapholita molesta* behavior [164].

Apart from olfactometry using a T- or Y-shaped apparatus, there are some other two-path structures that have been referenced in the literature that also involve the placement of test insects in a center point equidistant to both control and VOC-containing reservoirs. One such configuration separates the insect from the reservoirs by using conduits, and the vapors from each reservoir are pumped to the insect placement point. This has been cited in insect attraction assays regarding whole plants [68,165] or fruits [42], among others. Another interesting experiment with a nonconventional configuration is described in Ref. [166], in which the VOC reservoirs were underground sources, specifically pots containing soil that were planted with *Picea abies* L., and their influence on the attraction of *Otiorhynchus sulcatus* larvae was evaluated.

Another method is well olfactometry, in which the insect is placed over a platform that has two wells, and again, one well houses the VOC-emitting source and the other one houses the control. In this assay, the insect is released and then allowed to walk on the platform, and whether the insect approaches or moves away from a well is observed. It must be considered that in this assay, the VOC diffusion is passive, i.e., no pump is used for transporting the VOC to the insect. In this olfactometry method, the well can be below the platform level [67] or at the same height [39].

#### 5.3.3. Four-Path (or More) Olfactometry

In this type of arrangement, various containers with or without VOCs are placed equidistantly from a central area where the insects are released and connected to this part by individual conduits with equal lengths. This method has the advantage of allowing the use of emission sources and controls with replicates in the same assay to satisfy the statistical data distribution of the insects’ movement. Nevertheless, it is advisable to perform experimental repetitions with random positioning of sources and controls to avoid bias. This can be operated with VOC active diffusion from the reservoirs by pumping and with passive diffusion [1,43].

This type of olfactometry has been used to observe the attractive effect of *Trifolium platense* L. VOCs at different phenological stages on *Hylastinus obscurus* [167], in the evaluation of the choice of the aquatic beetle *Macroplea appendiculata* for exudates from relevant plants in various biological cycles by using a six-arm olfactometer filled with water [26]; the employment of a four-arm olfactometer with passive diffusion was used in assays of the attraction of *Trissolcus basalis* to buckwheat flowers, nonfloral organs, VOC extracts and synthetic compounds [1], and with active diffusion in other papers in which extracts and synthetic solutions of pheromones of *Alphitobius diaperinus* were assessed for repellency on individuals of the same species [29]. The repellent effect of an alarm pheromone associated with the aphid *Rhopalosiphum padi* [168] has also been referenced in the behavior of the beetle *Acanthoscelides obtectus* with grains of the bean host species, using healthy grains infested with other individuals of the same insect species or those treated previously with semiochemicals [169].

#### 5.3.4. Wind-Tunnel Assay

In this type of experiment, the odors are actively conducted through a chamber in which the insect is located in a controlled manner, with the purpose of generating an odor plume with uniform distribution and then evaluating the insect behavior. These assays can be used for assessing both flying and walking insects [14]. Some examples using these methods include the analysis of changes in the behavior of parasitoids related to *Brassica nigra* when exposed to emissions of plants infected with a bacterial phytopathogen [170], in the monitoring of parasitoid choice for plants in normal conditions or when plants are infested with different herbivores [38,170], and in the research on the specificity of *Myzus persicae* aphids to tobacco plants, including both generalist individuals and a subspecies specialized in tobacco plants [171]. In studies related to lepidopteran pest species, several examples are described such as the evaluation of responses of *Plodia interpunctella* to volatiles emitted by both healthy and fungus-infected wheat grains [67], the study of sex pheromone tracing by *Heliothis virescens* in an environment full of VOCs coming from plants under distinct conditions [172], the response of *Cadra cautella* to blends with different sex pheromone ratios [173], the assessment of the behavioral effect of attracticide formulations (i.e., a mixture of pheromones with insecticide) on males of *Choristoneura rosaceana* and *Pandemis pyrusana* [174], and the attraction testing on parasitoid *Glypta haesitator* exposed to volatiles related to its host, the moth *Cydia nigricana* [175]. 

### 5.4. Non-Choice Assays

In this type of biological assay, the VOC-emitting source is located within the same reservoir as the test insect at the beginning of the experiment. No-choice assays have been applied mainly in the research of biological control agents, as in the cases determining parasitoid specificity (preference) to pest species eggs [176,177]. Nevertheless, similar experiments have been performed in which, instead of using eggs as stimulus, a paper impregnated with semiochemicals or natural source extracts is offered to an insect, with the aim of observing the diffusion or contact effects of components presented in the paper, employing distinct configurations according to the experiment scope [178]. Several examples can be cited such as the study of those semiochemicals with the potential to affect the oviposition preference of the beetle *Acanthoscelides obtectus* on beans (being this beetle a pest of beans) [169], the effect of essential oils from *Achillea* spp. on *T. castaneum* [179], the insecticidal activity of diverse VOCs extracted from *Cymbopogon* and *Eucalyptus* on the same beetle [180], the response of the aphid parasitoid *Aphidius colemani* Viereck to pheromones obtained by extraction for determining courtship behaviors [181], and the parasitoid *Psyttalia concolor* response to specific volatiles emitted by host insect-damaged plants [182]; in addition, the analysis of repellent activity of extracts obtained from the beetle *Liposcelis bostrychophila* on the control of the same species has been evaluated [183].

### 5.5. Audiovisual Analysis

Experiments that evaluate behavior can be used in conjunction with video tracking, which allows for the recording of movements and actions of insects that may go unnoticed with direct visual inspection to be analyzed later in fuller detail. Among its advantages, is that it facilitates assay automation, which implies the possibility of markedly decreasing human interference during the assay execution, in addition to conferring evidence about the experimental development and allowing the evaluation of the same anytime.

This aid has been used in a very wide variety of studies. Citing some few interesting examples, audiovisual analysis has been employed for the determination of the repellent activity of essential oils on mosquito *Culex pipiens* from the interpretation of both visual and audio records [184]; in the differentiation of responses from *Myzus persicae* aphid generalists and those specialized in tobacco plants when exposed to both host and nonhost plants [171]; in the recording of sexual deception in male pollinating wasps using the orchid *Caladenia plicata* when dummies impregnated with a mixture of semiochemicals from the said flower were offered to the insects, with the observations of sexual behavior being comparable to that exhibited for the same during pollination [21]; for tracing changes in the feeding patterns of *Hylobius abietis* in pine seedlings treated with methyl jasmonate, performing a prolonged recording at defined hours [24]; and to follow the disturbances in interactions of *Tetranychus evansi* with its predator *Phytoseiulus longipes* under visual, vibratory and olfactory stimuli, both separately and in combination [39].

### 5.6. Sensor-Based Detection of Semiochemicals

In addition to instrumental techniques and behavioral assays, sensorics has also contributed to the development of different tools for the detection of bioactive semiochemicals on insects by means of chemo, bio and nanosensors selective toward semiochemicals. A recent review published by Brezolin et al., (2018) described the features and important details for the correct uses and scope of sensor-based detection of semiochemicals [185]. In the case of chemosensors, it is mentioned that electrochemical [e.g., potentiometric, conductimetric, or amperometric], piezoelectric [e.g., quartz crystal microbalance (QCM) and surface acoustic wave (SAW)], and optical [e.g., surface plasmon resonance (SPR)] sensors can be used for semiochemical detection. However, the sensitivity, selectivity, and response time are not comparable to the olfactory systems of living organisms [185,186]. From this fact, the evaluation of molecular structures involved in odor detection [i.e., hydrophobic proteins known as odorant-binding proteins (OBPs)] is the most common practice to discover/develop biosensors for specific/selective detection of semiochemicals [187]. 

Due to the significant evolution on the molecular interpretation of insect olfaction and chemoreception [187], OBPs are considered as the central molecular targets in a recently coined approach called “reverse chemical ecology” [188], constituting another convenient, reliable and efficient procedure for screening volatile semiochemical candidates [189]. As shown in Figure 1, this approach starts with the identification and expression of OBPs genes from target insects using bioinformatics and molecular biology-based tools. In this regard, the OBPs genes can be largely selected by RNA sequencing (RNA-Seq) when they are considerably enriched in the antennae of target insects compared with other organs (e.g., legs). This identification is often validated by analyzing the expression of identified genes through quantitative polymerase chain reaction (qPCR) [20]. Direct molecular cloning is the most commonly used approach for OBPs identification, comprising currently three steps such as the design of degenerate primers from conserved protein sequences, subsequent fragment amplification, and full-length sequence assembling through rapid amplification of cDNA ends (RACE) [190]. OBPs gene discovery is also possible using bioinformatics owing to the genome sequencing of various insects. Therefore, another strategy can be performed through a sequence comparison of the target insect genome or expressed sequence tags (ESTs) with available counterparts from other insects looking for OBPs-like orthologs [191]. Once the OBPs are identified in the target insect, such biomolecules are obtained from the original insect or recombinant organisms using protein extraction and purification protocols. Classical molecular methods, such as electrophoresis and sequencing, have also been used for OBPs characterization. The resulting OBP-containing protein extract (or purified OBP) is employed in OBP:ligand-binding and kinetic in-vitro assays for screening the test semiochemicals (previously identified from libraries or in house collections and precisely selected by in-silico methods, e.g., molecular docking [192]). Within the current investigations of OBPs, a few examples can be mentioned such as the measurement of ligand-receptor affinity for OBPs from the moth *Lymantria dispar* at different pH values [193] and the effect of free fatty acids on the active site proximities [194]; the recognition of an alarm pheromone in an aphid [168]; the assessment of the differences in an OBP in individuals of both sexes in two species of fruit flies by means of proteomics [195]; the identification of six OBPs of the dipteran *Carpomya vesuviana* and the measurement of their response to semiochemicals [80]; and the characterization of various OBPs of the moth *Plutella xylostella* present in different organs [196]. For honeybees, comparative studies of OBPs among different species have been carried out [197] as well as an investigation of the neonicotinoid effects on such receptors has been performed [198,199]. 

Similar approaches have also been used for emerging biosensor technology to detect semiochemicals using OBPs. Thus, the recent development of impedance biosensors for semiochemical detection is highlighted. These sensors were constructed from insect OBP immobilization on various materials, such as the honeybee OBPs on gold electrodes [200,201] or for oriental fruit fly (*Bactrocera dorsalis*) OBPs on polyethylene glycol membranes [186]. The most common application of nanosensors for semiochemical recognition is related to the cantilever modification used in atomic force microscopy (AFM). In this context, bioinspired cantilever nanosensors have better properties and advantages, which might promote the development of sensor arrays in the future to discriminate compounds at the femtogram level [185].

## 6. Statistical Analysis

The use of statistics in this field of knowledge is of crucial relevance and should be considered during the development of a study, starting with the experimental design. Correct planning allows not only the optimization of resource utilization but also provides guidelines for establishing the appropriate sample size and study suitability to answer a specific biological research problem in a manner that is impartially reviewed [202]. The present paper mainly considers statistical tools for data analysis which support the identification of VOCs with activity on insects. Within this topic, statistical analysis has been applied for assessing insect behavior and directed movements in olfactometers to better comprehend the effects derived from volatile sources and in VOC profiling by chemometrics, among other examples. The aid of statistical analysis in the execution of studies in this area is indicated by the fact that the data matrix can consider several variables and can contain large amounts of data, so that data management and interpretation are highly facilitated by using statistical tools.

### 6.1. Statistics in Olfactometry and Behavior

#### 6.1.1. Generalities

Olfactometry itself is a crucial assay for investigating chemical ecology interactions mediated by VOCs, because it provides information about insect behavior in response to a particular olfactory stimulus. Behavioral analysis must be performed with various repetitions in enough quantities according to the experimental design and scope to ensure sufficient data, and then the appropriate statistical model leads to successful interpretations. Although this notion is widely known, it is also common that related and necessary concepts of statistical analysis are misunderstood or even unfamiliar for many researchers, and then mistaken conclusions about assay results are made. After this, it is valid to consider some management steps for the statistics of olfactometry and behavioral studies, among which the following are the most important:

(1) Experimental design and scope: The utilization of robust experimental designs provides better accuracy for making conclusions about the results of an experiment, but it should be taken into account that incorrect interpretations of data by the researcher are always possible [203].
(2) Sample size and statistical power: Corresponding to optimal numbers of measurements, treatments and repetitions performed during the experimentation stage [204] should be sufficient to provide significant differences between the null hypothesis and alternative hypothesis [203].
(3) Pseudoreplication: Pseudoreplication is one of the most extensive problems in olfactometry and, subsequently, in associated reports [14,203,205,206]. It is strongly advisable to avoid this when possible. This consists of the use of non-independent experimental units during assays, generating a result bias and incorrect statistical conclusions. Often this is derived from incorrect experimental unit definitions (border effects, lack of randomization, poor variable control, etc.) or management (reutilization of the VOC source, insects, materials, and more), producing a “residual” effect amongst a unit and its replicates [205,206].
(4) Unbalanced data: This refers to data deficits due to unforeseen experimental events, such as difficulty measuring some variable, atypical or out-of-range values, etc., which produce an incomplete data matrix for analysis. In some cases, this can be corrected through two strategies: data deletion when experimental data are sufficient and insignificant information is lost or data filling for situations in which the data behavior is well known. Unfortunately, these approaches are not always feasible [206].

#### 6.1.2. Statistical Tools for Olfactometry Data Treatment

##### Parametric Tests

Corresponding to statistical tests, which can be used on a data matrix with a normal distribution (Gaussian) and whose data groups exhibit equal variances, certain parameters must be determined before using the test. Parametric tests can be applied directly on original matrix data or in those that achieved normal distribution after transformation, using precaution in the latter case for considering the changes in relationships among variables derived from conversion when conclusions are given [203]. The parametric tests are widely but sometimes incorrectly used in olfactometry assays, and their main advantage is that they have better statistical power for conducting a null hypothesis rejection compared to nonparametric tests, but the data requirements also restrict their utilization [206].

Among the most cited parametric tests, the *t*-test or Student’s test can be found, as well as the analysis of variance or ANOVA (and their derivative tests). The Student’s test has been applied for key VOC identification in samples with particular conditions, such as terpenes of *Pinus sylvestris* L. under induced defense [24], volatiles emitted by the roots of a plant colonized by an endophyte [43], in the search of compounds associated with a defective-status in seeds of California nuts [70] and coffee [108], for characterization of volatiles present in sexual glands from populations of *Spodoptera frugiperda* specialized in different crops [28], and in the validation of pheromone collection by using a GC-column fragment [45]. It has also been used for evaluating the responses of VOCs in EAG [37,67], in attraction and repellency assays [32,68], and in the comparison of an insect response to a natural VOC source compared to a synthetic mixture, among others.

ANOVA has been employed mainly in the determination of significantly different responses among groups of data matrices associated with biological activity results, which implies the creation of several replicates. ANOVA is usually complemented with Tukey’s HSD tests. Several examples using ANOVA are found in literature such as the evaluation of insecticidal activity of different essential oils on well-known pests, i.e., *Sitophilus zeamais* [106] and *Drosophila suzukii* [110], the attractive-repellent nature of selected plants on the coffee berry borer (*Hypothenemus hampei*) [163], the attractive-repellent effect of *Alphitobius diaperinus* abdominal gland extracts on individuals of the same species [29], the assessment of the aphid preference for emissions coming from *Chrysanthemum morifolium* and *Artemisia annua* [72]; and the characterization in EAG of the dose-response of floral VOCs on a parasitoid as a complement to the research on the effect of emissions on its fecundity [1].

##### Non-Parametric Tests

There are methods that do not demand a specific data distribution and are useful for a broader range of situations compared to the parametric tests but with less statistical power. These methods should be used when the underlying assumption of normal distribution of errors, required by the ANOVA test, cannot be achieved, and the ignorance of the distribution of errors does not allow applying a generalized linear model (GLM). Some examples of this type of test are the Friedman, the Fischer, the Mantel, the multiresponse permutation procedures (MRPP), the Mann-Whitney U, and the Kruskal-Wallis [203] tests. Some of these methods have been cited, for instance, the comparison of the composition of floral scents from two distinct orchid species that are exclusively pollinated by the same insect, *Eufriesea violacea* [76] by null hypothesis with MRPP. In addition, the differences in choice times for insects in olfactometry were evaluated by Friedman test [165]. The Mantel test has been used in the interpretation of the *Tanacetum vulgare* L. VOC chemotype and its correlation with geographical provenance [81], and the Kruskal-Wallis test for categorization of the intensity of aggressiveness of ants to their congeners when the latter were sprayed with certain VOC extracts, as well as for analyzing the flight behavior of individual *Heliothis virescens* moths to pheromones ventilated in a wind-tunnel [172]. 

##### Circular Statistics

The responses of walking insects to odors are largely comprised by directional nature. This is valid in olfactometry experiments as well as in field conditions. The direction of movement of an insect can be recorded as degrees or radians in a circle, which gives to this type of data a periodic nature. An accurate proposal for recording, measuring and analyzing directional responses under laboratory conditions is presented by Thiery and Visser (1987) [207]. In a broader context such as movement ecology, a proposal that could be promising for analyzing responses of insects to odor stimuli, under field conditions, is the use of multivariate angular regression models featuring angular and linear predictors [208].

##### General Linear Models (GLMs)

In many cases, the response variable of a behavioral assay cannot be assumed to be having a normal distribution of errors, as required for an ANOVA test. For example, a two-path olfactometer-based assay has two possible results; it has a binomial response variable, and therefore, a statistical model assuming a binomial error distribution is then required. In such cases, GLM is the appropriate analysis strategy, since it allows to define an adequate error distribution to be assumed from the exponential family of probability distributions [209]. GLMs describe the relationships between a dependent-variables matrix and an independent-variables matrix by means of a linearized model, which is analogous to a straight-line equation but using matrices (or vectors). Both the dependent and the independent variables are organized in separate vectors, in addition to the intercept and error-measures vectors that are added. The intercept and independent variable vectors can be consolidated by a matrix multiplication procedure to simplify the general model, resulting in an expression corresponding to the product of a slope vector with the independent-variables vector, and the remaining error vector becomes the general intercept vector [210].

The applications of GLM may include the following procedures for analysis: (1) the refinement of GC-MS data of essential oil VOC profiles prior to other statistical treatments, as one step of the multivariate curve resolution (MCR) procedure [211]; (2) assessment of the effect of specific VOC chemotypes on aphid colonization over *Tanacetum vulgare* L. [81]; (3) in determining the functional relationship of the response of *Costelytra zealandica* larvae to emissions coming from plants with or without an endophyte [43]; (4) for correlating the VOC profile from leaves with mechanical-damage as a function of time [71]; (5) and for associating the emissions of apple extracts with *Cydia pomonella* displacement with linear olfactometry [5].

### 6.2. Multivariate Analysis

There is not a defined consensus about the techniques for multivariate analysis which is a consequence of the immense variety of software, algorithms and statistical operations available, which sometimes results in difficulty in selecting the appropriate method for data processing [212]. Despite this, most analyses involve procedures that can be organized into four general steps as follows.

(1) Conditioning: Multivariate analysis is strongly scale dependent, and because of this, it is common that the data matrix be conditioned to ensure a convenient scale, misinforming variations are systematically reduced, noise is reduced [213], information among samples is aligned, remaining values are introduced, and data is filtered. For this purpose, it is possible to use several normalization methods [202].
(2) Pretreatment: This usually refers to conditioned data normalization to ensure comparability among samples or groups, and consistency must be maintained in experimental design and research work objectives [202,212].
(3) Treatment: This consists of performing the analysis to detect and identify general discrimination patterns among samples or groups, and the application of probabilistic models that determine the response, with statistical significance, to a factor under investigation [212]. For this work, descriptive models and classification models can be employed [213].
(4) Validation: The final step, validation, consists of the analytical characterization of a marker, i.e., a VOC that can be associated with a group defined by characteristics or properties in absolute terms to find the corresponding differences among sample groups and controls and corroborate the marker in question [212].

#### 6.2.1. Descriptive, Exploratory or Discriminating Models

These models are tools that analyze a data matrix and evaluate the repeatability of measures by sample grouping according to the shared degree of similarity. This implies that the values or groups with significant differences are notably separated, allowing the easy and quick identification of atypical values in this manner. From these techniques, self-organizing maps, principal component analysis (PCA), and hierarchical clustering analysis are among the most recognized methods [213,214].

PCA is a mathematical tool that aims to symbolize the present variations on a data set by using a reduced number of factors [214], i.e., PCA contracts the number of original variables to fewer ones denominated as principal components, which are linear combinations of initial factors [213,215]. The results of PCA can be inspected visually by means of scatterplots in 2D or 3D that make it easy to verify the individual data groupings or discriminations, although the results can also be evaluated by using factor loading tables that describe the results in numerical terms [214]. PCA is one of the most popular descriptive models because it is often employed in plant VOC profiling, for example, in volatiles emitted by flowers and nectar [69], the leaf interface [63], fresh fruit [109], coffee [108], essential oils [99], and more. This method has also helped in the investigation of changes in VOC emission patterns in plants directly affected by herbivory, as in the case of *Dittrichia viscosa* attacked by *Myopites stylatus* [73], in profiles comparing healthy and mechanically damaged California nuts (*Juglans regia* L.) [70], in broccoli infested with the *Delia radicum* fly larvae [79], and in the analysis of herbivore response changes to a plant under particular conditions, such as in the presence of endophytes [43] or during induced chemical defense [24].

Non-metric multi-dimensional scaling (NMDS) is an ordination technique suitable for analyzing a wide variety of data. NMDS involves few underlying assumptions and any distance measurement can be used. Unlike most ordination methods, a small number of axis in NMDS are selected by the user before analyzing the data; while in most other methods a big number of axes are calculated, few are visualized and the others remain as hidden axes of variation. Additionally, although most ordination methods obtain a unique solution, the NMDS is a numerical procedure searching for an iteratively-convergent solution, so the algorithm stops if an acceptable solution is reached. Owing to the multiple solutions that are produced, somewhat different ordinations can be obtained [216]. Despite the fact that this technique is slower than others, and in some cases fails to find the best solution due to being stuck in local minima, the increase of computing power has widely overcome these problems. This technique has been used to visualize and analyze plant–insect interactions such as the change in the emission of VOCs by *Phaseolus lunatus* in response to herbivory [217], the effects of volatile emissions of asparagus on its associated early season pest species (e.g., asparagus miner) [218], the differential attraction of pollinators by monkey flowers [219] and asclepiads [220], and the differential attraction of *Spodoptera littoralis* to five host plant species [221].

On the other hand, the HCA is a method that explores the sample distribution in groups by representing a hierarchy, which is shown in a dendrogram that consists of a tree-shaped scheme that displays the samples and exhibits their respective relationships and organization [214]. In contrast to the PCA, the individual data are not explicitly shown in the dendrogram because the hierarchy is calculated from the sample dataset. HCA has been used for assessing VOCs from cultured plants, such as loquat (*Eriobotrya japonica* Lindl.) [109] and rice (*Oryza sativa* L.) [65], for discriminating the origin of their varieties, which represents supporting information for the identification of cultivars with higher infestation potential for particular pests.

#### 6.2.2. Classification Models

These models are based on the specification of defined data classes by means of a property set, with the objective of evaluating the feasibility of sample assignation to any of these data classes. Some of these models are linear discriminant analysis (LDA), artificial neural networks (ANN), partial least squares discriminant analysis (PLS-DA), and more. The main effective application of classification models is when the number of variables exceeds the number of objects (samples) [213].

From the latter methods named above, the PLS-DA is one of the most popular in VOC profiling. This method calculates a regression model from a data matrix with instrumental measurements (e.g., chromatographic peaks VOC or concentrations) related to the sample class matrix in codified units (ones and zeros), predicting a corresponding zero or one value for each sample that is subsequently converted to a class marker using an optimized limit [213].

## 7. Conclusions and Perspectives

There is an extensive variety of techniques for evaluating VOCs with activity on insects, and more importantly, for those that play an important role in chemical ecology interactions. There are valid techniques for the steps involved in this kind of investigations, starting from VOC collection itself. Thus, instrumental, computational and biological methods for collecting data along with the corresponding interpretations aided by statistical methods can led to reliable concluding remarks.

Among current VOC collection methods, classic extraction techniques, such as solvent extraction or distillation processes, are convenient for obtaining significant amounts of compounds with chemical stability, with sufficient abundance, in forms easily recoverable from a matrix. However, for cases in which in-situ or in-vivo sampling is required due to technical difficulties, if performed under other conditions, VOC capture on sorptive materials is the most suitable alternative. In addition, organic synthesis is also an option for obtaining VOCs of some natural products with high purity in sufficient amounts for analysis; nevertheless, this can be challenging for compounds with some kinds of chirality.

Concerning VOC analysis, identification and characterization, the accessible methods are so much more diversified, each with their own advantages and limitations, and due to this, these methods are complementary to each other. The instrumental chemical analysis methods allow the molecular identification of different VOCs as well as their quantification with a high sensitivity, considering their often-low concentrations. Apart from this, molecular and behavioral biology indicate the effect or function of VOCs on test species, and such assays are the basis for determining the role of VOCs in chemical ecology interactions.

Finally, an unimaginable amount of data arises from collecting information using the previously named techniques, which then becomes difficult to interpret directly. For this, the statistical analysis tools play an important role in both data management and study, this being the reason for the increasing use in recent years. From statistical analysis, the elaboration of models from big data matrices and those with several variables has been facilitated, allowing the better recognition of their relationships and reach in chemical ecology.

## Figures and Tables

**Table 1:** Some stereospecific syntheses of insect-related semiochemicals.

Semiochemical	Source	Type	Key Conversions/Reactions	Ref.
bombykol	*Bombyx mori*	Sex pheromone	Ni- and Pd-catalyzed cross coupling reactions	[138]
(*Z*)-15-methyl-7-nonacosene and (*Z*)-17-methyl-7-hentriacontene	*Eurytoma maslovskii*	Sex pheromone	Grignard reaction	[139]
(3S, 5S, 6S)-tetrahydro-6-isopropyl-3,5-dimethylpyran-2-one	*Macrocentrus grandii*	Sex pheromone	cationic cyclopropyl-allyl rearrangement, diastereoselective alkylation and diastereoselective hydrogenation	[140]
(3*Z*, 9*Z*)-*cis*-6,7-epoxy-3,9-octadecadiene	*Ectropis obliqua*	Sex pheromone	regioselective dienol epoxidation (sequential ring-opening)	[131]
2*E*- and 2*Z*-homofarnesals (6:4 blend)	*Callosobruchus chinensis*	Sex pheromone	Ando’s *Z*-selective alkene synthesis	[132]
(*R*)-Lavandulyl propionate	*Dysmicoccus grassii*	Sex pheromone	Two-cycle enzymatic transesterification of racemic lavandulol using Porcine pancreas lipase	[46]
10, 14-dimethyl-1-pentadecyl isobutyrate	*Euproctis pseudoconspersa*	Sex pheromone	Evans’ methylation and C–C bond formation by Julia-Kocienski coupling and Wittig olefination	[129]
(+)-sitophilure	*Sitophilus oryzae* L. and *Sitophilus zeamais* M.	Aggregation pheromone	Enzymatic reduction using *S. cerevisiae*	[130]
(±)-frontalin	*Dendroctonus* genus	Aggregation pheromone	Double dihydroxylation, mono-cleavage, and acid-catalyzed intramolecular acetalation	[141]
4, 8-dimethyldecanal (four stereoisomers)	*Tribolium castaneum*	Aggregation pheromone	Organolithium-mediated reaction	[142]
